# Carbon Ion-Irradiated Hepatoma Cells Exhibit Coupling Interplay between Apoptotic Signaling and Morphological and Mechanical Remodeling

**DOI:** 10.1038/srep35131

**Published:** 2016-10-12

**Authors:** Baoping Zhang, Long Li, Zhiqiang Li, Yang Liu, Hong Zhang, Jizeng Wang

**Affiliations:** 1 Key Laboratory of Mechanics on Disaster and Environment in Western China, Ministry of Education, College of Civil Engineering and Mechanics, Lanzhou University, Lanzhou, 730000, China; 2 Department of Heavy Ion Radiation Medicine, Institute of Modern Physics, Chinese Academy of Sciences, Lanzhou, 730000, China; 3 Institute of Biomechanics and Medical Engineering, Lanzhou University, Lanzhou, 730000, China; 4 Key Laboratory of Oral Diseases of Gansu Province, Northwest University for Nationalities, Lanzhou, 730030, China

## Abstract

A apoptotic model was established based on the results of five hepatocellular carcinoma cell (HCC) lines irradiated with carbon ions to investigate the coupling interplay between apoptotic signaling and morphological and mechanical cellular remodeling. The expression levels of key apoptotic proteins and the changes in morphological characteristics and mechanical properties were systematically examined in the irradiated HCC lines. We observed that caspase-3 was activated and that the Bax/Bcl-2 ratio was significantly increased over time. Cellular morphology and mechanics analyses indicated monotonic decreases in spatial sizes, an increase in surface roughness, a considerable reduction in stiffness, and disassembly of the cytoskeletal architecture. A theoretical model of apoptosis revealed that mechanical changes in cells induce the characteristic cellular budding of apoptotic bodies. Statistical analysis indicated that the projected area, stiffness, and cytoskeletal density of the irradiated cells were positively correlated, whereas stiffness and caspase-3 expression were negatively correlated, suggesting a tight coupling interplay between the cellular structures, mechanical properties, and apoptotic protein levels. These results help to clarify a novel arbitration mechanism of cellular demise induced by carbon ions. This biomechanics strategy for evaluating apoptosis contributes to our understanding of cancer-killing mechanisms in the context of carbon ion radiotherapy.

Carbon ion irradiation (CII) is regarded as a cutting-edge technique in cancer therapy. Unlike conventional radiotherapy, CII can produce a Bragg peak of energy distribution, which can be shifted to focus on the tumor nidus, allowing precise control of the dose absorbed by tumor cells and tissues for accurate targeting and maximum elimination of tumor cells^1^. CII therefore exhibits superior physical dose distribution and a higher relative biological effectiveness (RBE) than conventional radiotherapy^2^. As the current best tool for external radiotherapy of inoperable tumors, it has an established role in the treatment of various localized, radioresistant tumors mediated by hypoxia that are near at-risk organs^3^. Theoretically, CII can induce cell apoptosis (CA) or programmed cell death (PCD)^4^,^5^,^6^. Different signaling pathways are activated and converge on apoptosis-related molecules to trigger cell death. CA was initially identified morphologically^7^. Subsequent investigations of CA have focused on its morphological features, molecular mechanisms, and the underlying biological behaviors of cells^8^,^9^,^10^,^11^,^12^, among which the clarification of subtle molecular mechanisms has been regarded as the primary objective^13^. However, there is a complex coupling interplay among morphological alterations, mechanical cues, and cellular functions^14^,^15^. In this context, one critical question regarding CA is how mechanical signals are sensed and interpreted through the molecular machinery that mediates mechanotransduction. Although much is known about how biochemical signaling can direct cellular behavior^16^, relatively few studies have been conducted to investigate the systematic coupling effect between cellular mechanical change and the activity of organized and composited signaling molecules in transduction pathways. There are several exceptions. For example, Bakal *et al*.^17^ described the presence of local signaling networks that may regulate cell shape and migration based on the morphological profiling of single cells combined with RNAi-based genetic screening technology. In addition, Wada *et al*.^18^ suggested that cellular morphology may play a crucial role in regulating the Hippo pathway. Although much progress has been achieved in recognizing the relationship between certain cellular morphologies and molecular processes, a quantitative understanding of the complicated coupling interplays between mechanical and morphological factors and signaling pathways remains unknown and should be addressed in future research^18^.

Based on the CII, the present study established a CA model based on investigations of different HCC and normal hepatocyte (NH) lines to determine the coupling interplay of apoptotic signals that are spatially and temporally associated with morphological and mechanical remodeling to control cell death. Accumulating evidence suggests that phenotypic changes inevitably and continuously occur during morphological and mechanical remodeling within a cell and are accompanied by alterations in mechanical properties^14^,^15^. Elegant studies have demonstrated that the innate mechanical properties of cells can act as novel biomarkers to discriminate differences between normal and cancer cells during the evolution of cancer^19^,^20^,^21^. Coupled to the apoptosis process is the presence of tight regulation of molecular signals, which ensures that cells undergo survival or death. However, external stimuli may effectively participate in cellular and sub-cellular activities, resulting in variations in cell growth, basic remodeling, mechano-signal transduction, and gene expression^16^. Therefore, we should seek to understand how these clues mediate the onset of basic coupling and remodeling to switch on or off the cellular machinery. Evidently, such couplings are critical for multicellular organisms to maintain tissue and organ structures. Biotype atomic force microscopy (AFM) provides a multifaceted platform to directly measure the mechanical parameters of a cell (such as cell elasticity, cell geometry and size, and the degree of cell surface roughness) and to study their responses to applied forces^22^,^23^. This procedure allows quantitative investigations of how mechanical mechanisms are sensed by cells, and how the cells respond to applied forces and orchestrate the apoptotic remodeling process, which is characterized by changes in cell deformation and molecular damage events. Consequently, this single-cell analysis technique is helpful in determining the mechanisms underlying CA.

The aim of this study was to investigate the changes in mechanical signatures and morphological phenotypes during CII-induced HCC apoptotic signaling to determine whether there is a correlative and coupling interplay between them. Furthermore, using theories from statistical physics, we propose a theoretical model to explain how the mechanical mechanism may control the budding process of apoptotic bodies (ABs) that accompany the modulation of apoptotic signaling. Our results help to explain the different CII-induced apoptotic responses observed in different HCC lines by relating the responses to mechanical phenotypic signatures. This evaluation of apoptosis based on biomechanics may facilitate the attainment of greater knowledge regarding cancer-killing mechanisms in the context of CII. Such knowledge may enable the expansion of previously described single biological strategies for cancer diagnosis and evaluation of the curative effect during therapy, especially in clinical hepatoma therapy, using CII.

## Results

### CII-triggered apoptotic signals change in HCC and NH lines

**Figure 1:**
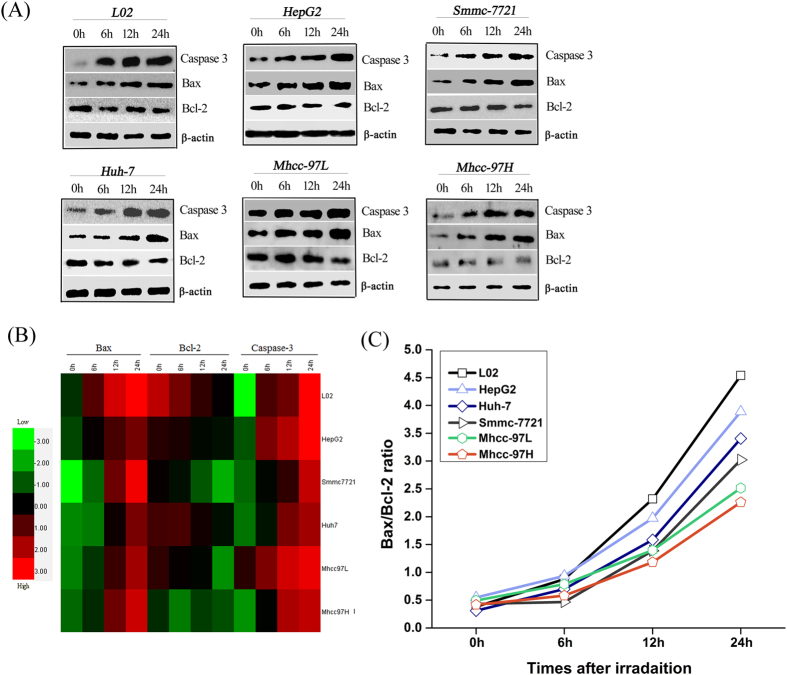
Quantitative alternation of apoptotic proteins in HCCs and NHs at different time points after CII. (**A,B**) IB analysis of the quantitative expression levels of key apoptotic proteins. (**C**) Distribution of the Bcl-2/Bax ratio.

An approach based on immunoblotting (IB) was established to determine whether apoptotic proteins mediate the signals that initiate CII-induced apoptosis and to understand how apoptotic proteins participate in basic remodeling within a cell. Figure 1A shows that CII upregulated the expression levels of caspase-3, increased Bax expression and decreased Bcl-2 expression in irradiated cells in a time-dependent manner. By contrast, increased Bax expression and no obvious changes in Bcl-2 expression were observed in the Mhcc-97H cell line after irradiation. Furthermore, hierarchical cluster analysis quantitatively revealed differences between the expression of apoptotic proteins in the different cell lines at different testing times. As shown in the heatmap in Fig. 1B, Bcl-2 expression was robustly downregulated in the irradiated cells relative to the nonirradiated cells, but the expression levels of Bax were upregulated in the irradiated cells relative to the nonirradiated cells. Bax and caspase-3 expression in these irradiated cells was gradually activated over time (0–24 h). However, Bcl-2 expression gradually decreased over time. The results also indicated that CII increased the Bax/Bcl-2 ratio (Fig. 1C). Accordingly, the change in the Bax/Bcl-2 ratio in the irradiated cells was more sensitive for forecasting cellular fate, and therefore this ratio can be regarded as an indicator to assess the progression of CA induced by CII.

### Quantitative analysis of the geometric changes in morphology during apoptosis

Various geometric parameters of the five irradiated HCC lines and one NH line were measured using cluster-cell and single-cell analyses. The physical parameters measured included the length, width, perimeter, thickness, cell projected area (CPA), and effective volume of the cells with CII doses of 0 and 2 Gy (Table 1). CII caused a remarkable decrease in all geometric parameters of the irradiated cells, and only the cell widths slowly changed (Table 1), implying that the change in morphological remodeling correlated with the shrinkage of HCCs and NHs.

### Single-cell profiling of precise topographic characteristics

**Figure 2:**
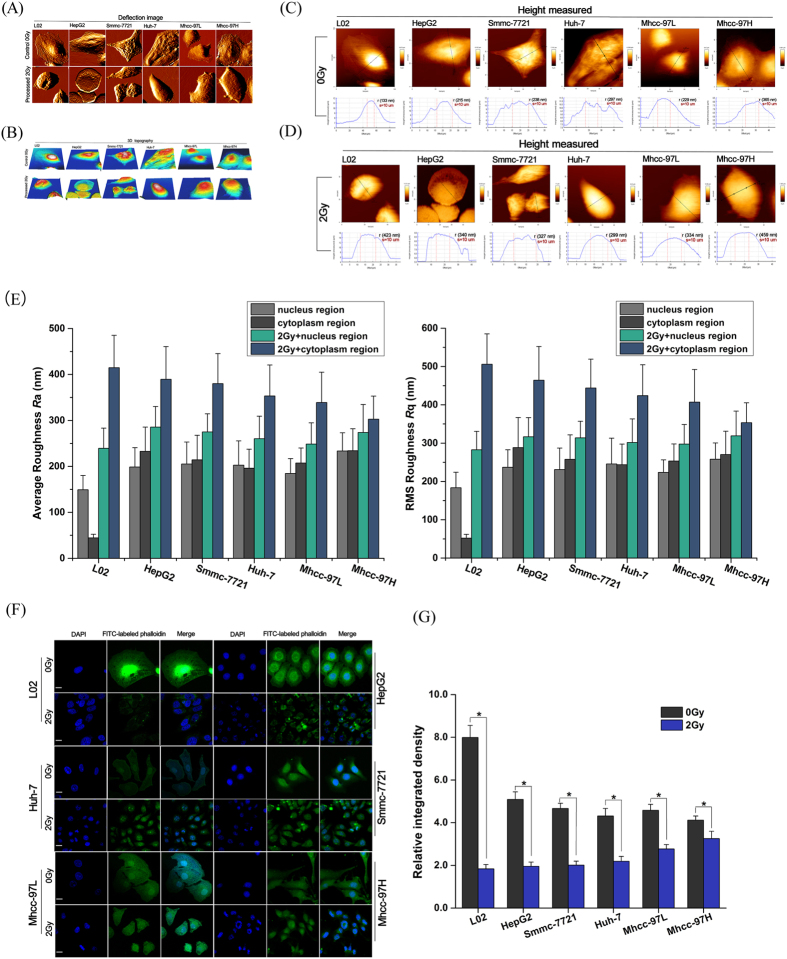
AFM micrographs of morphological changes in HCCs and NHs before and after CII at 24 h. (**A**) Deflection image. (**B**) 3D image. (**C–E**) Cross-sectional profiles revealing the similarity of the geometric size changes between cell height and surface roughness (*R*a and *R*q) in six cell lines. **(F)** Visualization of the cytoskeletal architecture in HCCs and NHs before and after CII. Scale bar, 10 μm. **(G)** Relative densities of the cytoskeleton distribution determined using ImageJ software for HCCs and NHs before and after CII. The mean ± SD is shown. *P < 0.05, Student’s t-test.

Figure 2A,B presents AFM micrographs showing the morphology of the irradiated and nonirradiated HCC and NH lines. The nonirradiated cells were characterized by a polygonal shape. The ultrastructure of the cell surface was homogeneous and displayed a gentle sloping decline from the apical zone of the nucleus to the limbic zone of the cytoplasm. The cell membrane was relatively smooth and intact, whereas the nucleus displayed continuous upheaval and plumping. After irradiation exposure, the cells shrank and became round. The plasma membrane was severely damaged and became rougher, exhibiting larger surface fluctuations and appearance of crimp on plasma membrane (Fig. 2A–E). To further elucidate the signature cell morphology on a nanoscale, the cell surface roughness was evaluated. The *R*a and *R*q changes in surface roughness in these cells revealed that the values of irradiated cells were larger than those of nonirradiated cells (Fig. 2C–E). These results confirmed that changes in cell surface roughness could serve as the most logical reflection of membrane surface remodeling during apoptosis.

Single-cell topography based on AFM analysis suggested that the NHs exhibited a neat actin filament arrangement before irradiation (Fig. 2A,B). Stress fibers in the limbic zone of the cytoplasm were arranged in tightly packed parallel striates that spread to the cell rim. In contrast to the NHs, the actin filament arrangement of the HCCs was chaotic. Stress fibers in the limbic zone were no longer in parallel striates. After irradiation exposure (Fig. 2A–D), the cytoskeletal structures of all HCC and NH lines were disassembled and indistinguishable, and the actin filaments were almost dissolved. In particular, several fine structures/units were observed, including certain irregular protuberances, small pores on the membrane, and fewer microvilli. The pseudopodial structure (lamellipodia) disappeared around the cell with a thin mesh-like layer of fibers.

### Cytoskeletal architecture disassembly enhances morphological remodeling

Based on immunofluorescence (IF) imaging, the CII-induced alterations in the actin cytoskeletal structure, their reorganization, and the cell nucleus in the HCC and NH lines (Fig. 2F) were compared with single-cell imaging using AFM. Abundant actin filaments were observed across the cell body in NHs and displayed a relatively organized orientation. These fibers presented a radial arrangement pattern that extended from the nuclear region. In particular, a clear filamentous perinuclear actin cap at the apex of the nuclear region was observed on the cytoplasmic surface. The actin filaments were organized in a more abundant cytoskeletal structure that consisted of unidirectional, parallel filaments throughout the cell body. By contrast, HCCs displayed fewer stress fibers at the basal surface, and most of the actin filaments were present in the filopodia and lamellipodia. The actin cap structures of the HCCs were undeveloped, but many short microvillus structures were present on the cell membrane surface. The nucleus was full and quasi-circular or elliptical in shape. F-actin mainly emerged from enriched vesicle-like structures in the HCCs after irradiation. In particular, the actin filaments randomly surrounded the nucleus without a common orientation, and the actin filament network condensed. The actin filaments were also similarly characterized by dissolution. Compared with nonirradiated HCCs, the integral cytoskeletal architecture following irradiation manifested as a random, disordered actin meshwork arrangement (Fig. 2F). However, the nucleus, as another radiosensitive organelle, became irregular, exhibited heteromorphic nucleus or microvoids, and presented nuclear pyknosis, nuclear fragmentation, and karyolysis phenomena. Quantitative image analysis revealed a significant decrease in the relative densities of the cytoskeleton post-irradiation (Fig. 2G).

### Cellular mechanical properties involved in CA

**Figure 3:**
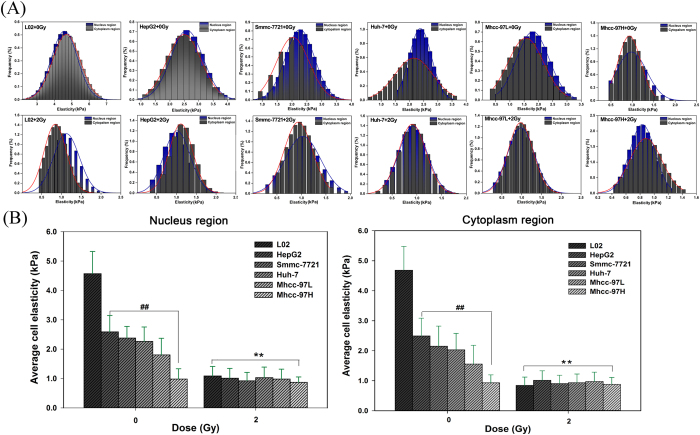
Distribution histograms of HCC and NH mechanical properties before and after CII. (**A**) Cell elasticity. (**B**) Averaged elasticity value. The mean ± SD is shown. ## and **P < 0.05, ANOVA. For mechanical properties, data collected from force spectroscopy curves were subjected to the Shapiro–Wilk normality test.

Cellular mechanical properties were distinguished from the nuclear and cytoplasmic zones using the single-cell mechanics method. The NHs showed a larger stiffness (elastic modulus, E) value than the HCCs (Fig. 3A,B). The elastic modulus of the NHs exhibited a larger range of variation with a normal distribution, whereas that of the HCCs displayed a narrower span of normal distribution. The order of the stiffness value of the nonirradiated HCCs was HepG2 >Smmc-7721 >Huh-7 >Mhcc-97L >Mhcc-97H. The nuclear zone in the HCCs exhibited a higher stiffness value than that in the cytoplasmic zone, but the stiffness of the cytoplasmic zone in NHs was significantly larger than that in the nuclear zone. Excluding the basal region (i.e., the coverslip substrate), the largest stiffness value was observed in the limbic zone of the NH cytoplasm. The softer region was located where the margin of the cytoplasm nearly extended to the apical area of the nuclear zone in the NHs. For the HCCs, the cytoplasmic margin that nearly extended to the apical area of the nuclear zone displayed greater stiffness (Fig. 3A). By contrast, an evident decrease in stiffness in the nuclear and cytoplasmic zones of the irradiated cells was observed compared with that of the nonirradiated cells. Certain similar changes in stiffness were observed in all irradiated cell lines, regardless of the partitioning into the nuclear and cytoplasmic zones.

### Correlation between the morphological phenotype and mechanical signatures during apoptotic signaling induced by CII

**Figure 4:**
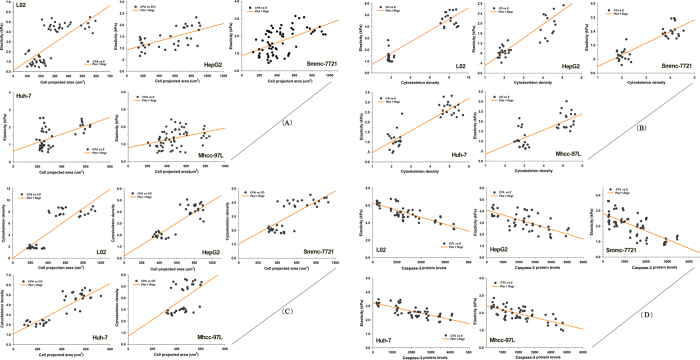
Correlations among variable parameters during apoptotic remodeling of HCCs and NHs. (**A**) Cell shape vs mechanical properties. In detail, CPA *vs* E, in L02, r = 0.642, *P* = 0.00 < 0.01; in HepG2, r = 0.619, *P* = 0.00 < 0.01; in Smmc-7721, r = 0.519, *P* = 0.00 < 0.01; in Huh-7, r = 0.564, *P* = 0.00 < 0.01; in Mhcc-97L, r = 0.355, *P* = 0.005 < 0.01; (**B**) Cytoskeleton density vs mechanical properties. In detail, CD *vs* E, in L02, r = 0.872, *P* = 0.00 < 0.01; in HepG2, r = 0.790, *P* = 0.00 < 0.01; in Smmc-7721, r = 0.876, *P* = 0.00 < 0.01; in Huh-7, r = 0.866, *P* = 0.00 < 0.01; in Mhcc-97L, r = 0.724, *P* = 0.00 < 0.01; **(C)** Cell shape vs cytoskeleton density. In detail, CPA *vs* CD, in L02, r = 0.841, *P* = 0.00 < 0.01; in HepG2, r = 0.784, *P* = 0.00 < 0.01; in Smmc-7721, r = 0.700, *P* = 0.00 < 0.01; in Huh-7, r = 0.568, *P* = 0.00 < 0.01; in Mhcc-97L, r = 0.501, *P* = 0.003 < 0.01; (**D**) Caspase-3 protein levels vs mechanical properties. In detail, CPLs *vs* E, in L02, r = −0.811, *P* = 0.00 < 0.01; in HepG2, r = −0.784, *P* = 0.00 < 0.01; in Smmc-7721, r = −0.714, *P* = 0.00 < 0.01; in Huh-7, r = −0.747, *P* = 0.00 < 0.01; in Mhcc-97L, r = −0.701, *P* = 0.003 < 0.01.

Although a large amount of evidence supports potential links between cell morphology, mechanical signatures, and apoptotic signals, the systematic coupling mechanism by which apoptotic remodeling is linked remains unclear. Based on the available data, we analyzed the relationship between CPA, E, cytoskeletal density (CD), and caspase-3 protein levels (CPLs) and all irradiated cell lines using Pearson’s product–moment correlation test. Figure 4A–D showed that except for Mhcc-97H cells, CPA and E, CD and E, and CPA and CD were positively correlated with HCCs. By contrast, E and CPLs were negatively correlated. These findings suggest that the apparently close relationship among the cell microstructure, actin cytoskeletal organization, cell mechanical properties and apoptotic protein expression is likely to play a crucial role in the onset of apoptosis.

## Discussion

Apoptosis, the regulated destruction of a cell, is a complicated process. The decision to die relies on the activity of many genes that influence the likelihood of a cell to launch its self-destruction program^24^. The coordinated activation of different signaling molecules is essential for proper execution of the apoptotic cascade. Approximately two-thirds of the identified caspase molecules have been suggested to function in apoptosis^25^,^26^. In most cases, irradiated tumor cells have been demonstrated to undergo apoptosis^27^,^28^. Many studies have explored the molecular mechanisms underlying the killing effect of CII^29^,^30^. However, how the systematic coupling associated with morphological phenotype and mechanical signatures regulates CII-induced apoptotic remodeling and how heavy ion radiotherapy evokes the killing effect in different tumor cells remain unclear. Therefore, the coupling interplay that mediates apoptotic signals in HCC lines under CII must be carefully investigated.

Our data indicated that cellular demise occurred through the activation of caspase-3 apoptotic signals within 24 h after irradiation, and the Bax/Bcl-2 ratio clearly showed a monotonic increase during apoptosis, which can be interpreted as an index to assess the progression of HCC apoptosis (Fig. 1A–C). Accumulating evidence suggests that caspases, a family of endoproteases, provide critical links in cell regulatory networks that control cell death^31^. The activation of apoptotic caspases results in the inactivation or activation of substrates, and the generation of a cascade of signaling events permits the controlled demolition of cellular components^32^,^33^. In particular, caspase-3 is important or essential in other apoptotic scenarios in a remarkable cell type- or death stimulus-specific manner^34^. Recently, A Tomiyama *et al*.^27^ demonstrated that CII-induced caspase-dependent apoptosis was initiated in human glioblastoma cell lines. Our results are also consistent with the findings of Fei Ye *et al*.^29^, who elucidated the mechanism of brain damage after exposure to CII and observed Bcl-2 family protein activation followed by activation of caspase-3. Unambiguous answers regarding the apoptotic mechanisms have been deciphered by caspase-mediated pathways to integrate death signals generated by a chain of molecular events, resulting in DNA damage, cytoskeletal demolition, and endoplasmic reticulum stress^35^. The influence of radiation cues to modulate the Bax/Bcl-2 ratio is regarded as an important determinant of its pro-apoptotic or anti-apoptotic role^36^. These findings highlight that the activation of caspase-3, Bax and Bcl-2 expression actively initiates signaling events within 24 h that are important for the CII-induced apoptotic response during the death of different HCCs.

Morphological and mechanical remodeling drives ongoing apoptosis to determine an underlying, conserved, and endogenous cell death program. It is reasonable to propose that a proper understanding of morphological remodeling will clarify the mechanism underlying the coupling interplay. An evident change in geometric parameters is characterized by morphological remodeling during CII-induced apoptosis. For example, the length, width, perimeter, thickness, CPA, and effective volume decreased (Table 1), whereas the surface roughness increased (Fig. 2E). Following the volume reduction in cells, if the cell height decreased, then the CPA decreased according to simple geometric rules. The measurements demonstrated a similar relationship among these geometric parameters, which reflects this loss of the geometric configuration of the cell shape during CII-induced morphological remodeling. However, the increased cell surface roughness implies a faster loss of cell volume than the area of the cell membrane during CII-induced cell shrinkage. All of these measured geometric parameters on the fine surface ultrastructure and gross morphological features of the cells are closely related. Intuitively, measurement of size parameters post-irradiation can help to accurately depict the change in micromorphology during apoptosis to better reflect the real-time physical state of the cell. Therefore, CPA was selected as a cross-sectional marker to assess morphological remodeling during apoptosis.

To further verify whether mechanical phenotype signatures are involved in the apoptotic coupling process, we focused on single-cell topography and cellular mechanical properties based on cytoskeletal alterations during mechanical remodeling. Our results demonstrated that CII caused the thin mesh-like layer of the fiber matrix to disappear and the cytoskeletal fibers to undergo rearrangements; thus, the mechanical properties of the cell could be radically altered (Figs 2A and 3). This finding shows that the differences in the mechanical properties of the cells lie in the fiber packing and the structural assembly of the cytoskeleton rather than the inherent cell shape. In this study, the fiber arrangement and the density of the fiber distribution jointly determined the cell stiffness. In particular, the orientation of the mesh structure of the interlaced stress fibers in the plasma membrane might also have increased the stiffness. Moreover, single-cell mechanical results revealed that the irradiated cells exhibited a decreasing tendency to exhibit the changes cell stiffness (Fig. 3A–C). In fact, the mechanical properties both NHs and HCCs depend on the conversion of extracellular cues into intracellular cytoskeletal responses. The viscoelastic cytoskeleton provides continuous mechanical coupling throughout the cell that changes during cytoskeleton remodeling. Such mechanical actions may conduct mechanical stresses from the membrane to internal organelles^14^,^37^. Evidence suggests that the mechanical properties of the cell are essential for generating responses because they determine the extent of cellular deformation or damage in response to the applied forces^38^,^39^.

In seeking consensus, the changes in the flexibility of cell structure may depend on the cytoskeleton. The actin cytoskeleton is a major eukaryotic cellular component because actin protein molecules undergo a continuous process of rapid polymerization and depolymerization to promote the morphological plasticity of living cells^40^. Furthermore, actin can be assembled and disassembled in response to a signaling cascade as a starting point in the pathway, which is related to the dynamic properties of the cytoskeleton to further modulate cellular activity^41^,^42^. In this study, we were interested in the impact of the disruption of the actin cytoskeleton on CII-induced apoptosis and whether the cytoskeletal structure is affected in different HCC and NH lines, which would alter the mechanical properties of the cell. After irradiation exposure, the F-actin cytoskeletal network architecture was condensed, and the actin filaments were similarly characterized by depolymerization and dissolution (Fig. 2F,G). Analogous investigation of apoptotic changes induced by okadaic acid result in disruption of the F-actin cytoskeleton, and activation of caspase-3^43^. Remarkably, the effect of actin cytoskeletal damage during the apoptotic caspase-initiated cascade is how cooperate to orchestrate actin dynamics within living cells, and how specifically cleaved by caspases^41^, which, in turn, results in the demise of the actin cytoskeleton. In support of links between actin and caspase-3 activation as the involvement in cytoskeletal disruption to elicit characteristic membrane blebbing and morphological changes, the pioneering findings suggests that caspase-3 can generate fragment of gelsolin^44^. Attributed to actin severing protein gelsolin was identified as a substrate for caspase-3, and caspase-cleaved gelsolin can sever actin filaments *in vitro*^45^. As accompanied by a reduction in F-actin content, the destruction of the actin cytoskeleton is sensitive to caspase-mediated damage as a consequence of apoptosis, and dynamic damage to the cytoskeleton appears to modulate downstream signaling events^46^,^47^. Clearly, the underlying molecular mechanism of gelsolin fragmentation induced by caspase-3 contributes to actin cytoskeletal collapse and nucleolysis^48^, which mediate, in part, the morphologic changes of apoptosis and also a mechanistic role for actin-severing protein in the apoptotic caspase-initiated cascade. This process requires cells to combine a signaling pathway involved in cell survival with the remodeling of the actin cytoskeleton. Therefore, cytoskeletal remodeling in response to radiation stimuli is a fundamental process in dying apoptotic cells. A less dynamic cytoskeleton would be unable to respond effectively to such cues. In this scenario, the pathway both regulates and is regulated by actin dynamics^41^, which appears that cell fate is associated with the stability of the cytoskeleton, with a close link between the dynamic properties of actin and apoptosis.

**Figure 5:**
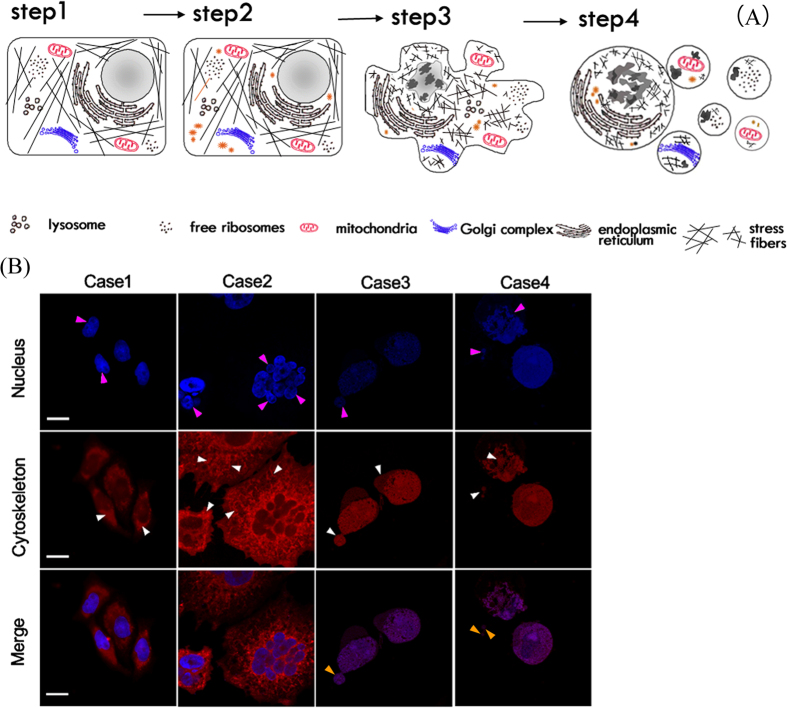
Morphological remodeling during CA. (**A**) Schematic diagram. **Step 1**: a healthy cell. **Step 2**: an unhealthy cell with activation of suspicious molecules. **Step 3**: an apoptotic cell with a series of typical morphological characteristics, including nuclear condensation, nuclear pyknosis, chromosomal DNA fracture, cell shrinkage, cytoplasmic blebbing, and cytoskeletal collapse. **Step 4**: continuation of apoptosis along with cellular budding and apoptotic body formation, resulting in cellular disintegration into many small apoptotic body segments (yellow arrows). These small bodies are engulfed by the macrophages *in vivo*. Apoptosis completes the evolution cycle accordingly. (**B**) Experimental observations of typical morphological changes in apoptotic HepG2 cells post-irradiation, including chromatin condensation, nuclear pyknosis, karyorrhexis, fragmentation (pink arrows), plasma membrane blebbing, a disorderly arrangement and dissolution of the cytoskeleton, an enlarged F-actin filament texture, enriched vesicle-like structures, and cytoskeletal collapse or remodeling (white arrows). These changes lead to cellular budding and apoptotic body formation (approximately 0.2–2 μm). DAPI-labeled nuclei (blue) and rhodamine phalloidin-labeled cytoskeleton (red). Scale bar, 10 μm.

Apoptosis is a multistep, evolutionarily conserved process that is characterized by the succession of signaling molecules and morphological changes that result in the selective and highly regulated dismantling of cell structures^43^. Typically, morphological hallmarks of apoptosis are characterized by shrinkage of the cell and nucleus, as well as condensation of nuclear chromatin into sharply delineated masses that become marginated against the nuclear membranes. During cytoplasmic blebbing, the nucleus progressively condenses, along with karyorrhexis, the appearance of depolymerization, and disintegration of the actin cytoskeleton. Afterward, cellular budding occurs in a process whereby the extensions separate and the plasma membrane seals to form a separate membrane around the detached solid cellular material. Consequently, ABs are crowded with closely packed cellular organelles or fragments of nucleus^49^,^50^,^51^. However, those fine structures/units contain membranes, mitochondria or other smaller organelles that are well preserved inside the bodies (Fig. 5A,B). To refine our understanding of conventional apoptotic events, the underlying biomechanical mechanism of the onset of apoptosis and the biophysical mechanism of the formation and cellular budding process of AB should be further investigated. We propose a theoretical model to explain how the mechanical mechanism may control the budding process of ABs associated with the progression of molecular pathways.

Our theoretical analyses are the first to elucidate the biophysical mechanism of the formation and budding of ABs during apoptotic remodeling. As shown in Fig. 5A, the apoptotic process must accompany AB budding, which can be clearly observed inside and outside the irradiated HCC shown in Fig. 5B. The molecular mechanism can explain most of the CA events but cannot answer the question of how the ABs are formed and discharged from cells. In this study, we hypothesize that during the CA process, a large number of free small molecules, biopolymer fragments/units and organelles are produced, depolymerized, and detached, thereby crowding the cellular environment. Under such conditions, the entropic depletion effects can lead to phase separation in the microsystem, which consists of large and small nano/microparticles^52^,^53^,^54^,^55^. Therefore, large ABs can be pressed against the cell membrane during CA. Based on an idealized theoretical model shown in Fig. S1, this driving force can be estimated as ~*F*_d_ = 4*πk*_B_*Tρ*_s_*R*_c_*R*_b_*R*_s_(*R*_c_ − *R*_b_ − *R*_s_)/(*R*_c_ − *R*_b_)^2^, where *k*_B_ is the Boltzmann constant, *T* is the temperature, *ρ*_s_ is the density of the free small molecules, and *R*_c_, *R*_b_, and *R*_s_ are the effective radii of the cells, ABs, and small molecules, respectively. Under certain conditions, this driving force can overcome the resistance of membrane bending with effective force, ~4*π*(*κ*/*R*_b_ + *γR*_b_), releasing the ABs from the cell through budding, where *κ* and *γ* are the bending stiffness and surface energy of the cell membrane, respectively. A detailed estimation of various parameters in this analysis shows that ABs with sizes from 0.5 μm to 1 μm can be squeezed out of the cell, which is consistent with the experimental observations shown in Fig. 5B.

Ionizing radiation often induces cells to undergo apoptosis in a synchronous manner; thus, apoptosis can be divided into biochemical signals, morphologic hallmarks and mechanical phenotypic phases. We next explored the method by which apoptotic remodeling associated with morphological phenotypes and mechanical signatures evokes different killing effects in HCCs during CII radiation. Based on the correlation analysis, the results in Fig. 2F show that the cytoskeleton of irradiated cells mainly formed by actin filaments exhibited a dissolved state, and its density significantly decreased after irradiation. These changes in cytoskeletal fibers are coincident with the evolution of the morphological phenotype and mechanical signature of cells undergoing apoptosis, as is clearly shown in the correlation in Fig. 4B. Furthermore, Fig. 4A,C,D illustrate that the representative parameters of cellular morphology, mechanical properties, and signaling molecular are highly correlated. Our data suggest that the coupled interplay during CII-induced apoptosis is conducted by the coordinated activation of apoptotic molecules and ubiquitous mechanical coupling, which results in a complex cascade of events that link the initiating radiation stimuli to the final demise of the cell. This relationship appears to have been maintained in divergent eukaryotic systems.

**Figure 6:**
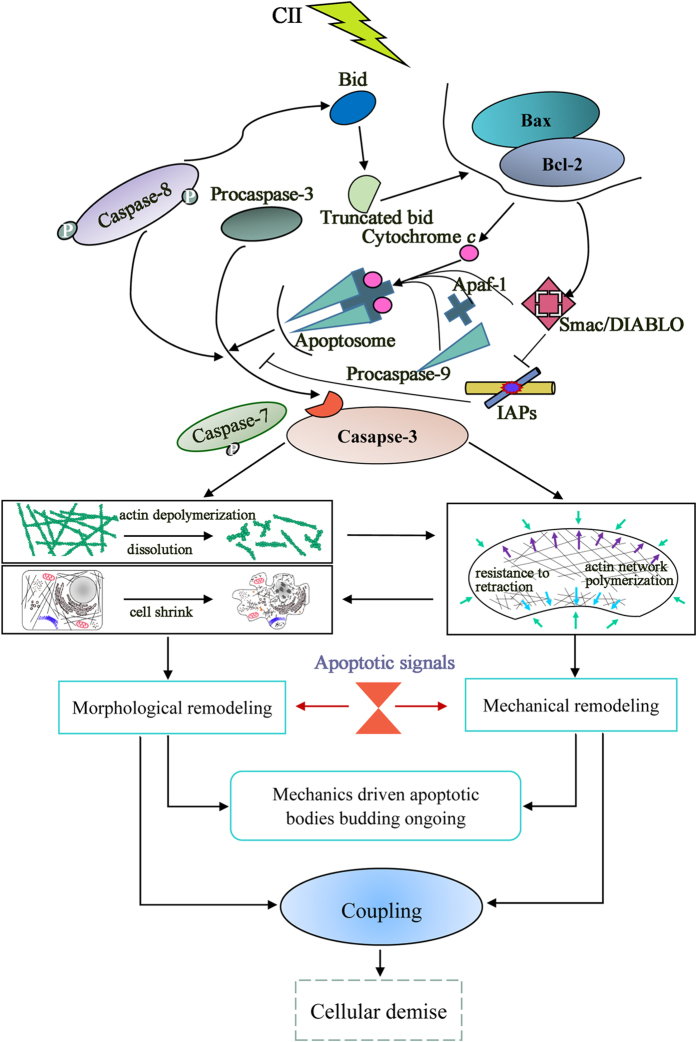
Coupling interplay mechanism during CII-induced HCC apoptotic remodeling: arbiters of cell survival or demise. This study has elucidated that a tight coupling interplay underlies CII-induced apoptotic remodeling. Firstly, ionizing radiation stimuli triggers activation of the apoptotic signaling pathway (Bcl-2 family proteins). Secondly, in the committed and effector phase, the molecular executioner caspase-3 becomes completely activated, committing the cells to evoke apoptotic changes. This phase is also accompanied by alterations in cellular mechanical properties, and the mechanical signals participate with the signals of specific cellular events to generate positive coupling feedback to modulate the apoptotic response process. The budding process of ABs is a remarkable example of this control by mechanical factors. Thirdly, a tight coupling interplay mechanism directs the cell toward survival or demise (Fig. 6). In the final stages, the ABs are rapidly phagocytosed by macrophages and parenchymal cells and degraded. Apoptosis completes the cycle.

The present study demonstrated that a tight coupling interplay between morphological and mechanical remodeling and signaling molecules, which are interwoven with the progression of CA. These observations on representative parameters of our data were identified to characterize the molecular process and morphological and mechanical remodeling of irradiated HCCs. Statistical analysis of these parameters showed a strong correlation. Theoretical analysis justified that AB budding is driven by the entropic depletion effect in addition to the molecular mechanism. Accordingly, such a tight coupling interplay between the molecular pathways and morphological and mechanical remodeling modulates a novel arbitration of cellular demise in CII-induced HCC apoptosis (Fig. 6). This strategy of evaluating apoptosis based on biomechanics may greatly improve our understanding of the cancer-killing mechanism in the context of CII. It may also help to expand previously described single biological strategies for cancer diagnosis and evaluations of curative effects during cancer therapy, particularly during the treatment of hepatoma with heavy ion radiotherapy.

## Materials and Methods

### Cell lines and cell culture

Five HCC lines, HepG2, Smmc-7721, Huh-7, Mhcc-97L, and Mhcc-97H, and a control NH line, L02, were obtained from the China Center for Type Culture Collection (Shanghai, China). All cell lines were cultured in Dulbecco’s Modified Eagle Medium (DMEM; Gibco, USA) supplemented with 10% fetal bovine serum (FBS; SH30084, Thermo Fisher Scientific, Rockford, USA), 1% antibiotic-antimycotic solution (15240-096, Invitrogen-Gibco, Karlsruhe, Germany), and 2 mM L-glutamine (G7513, Sigma, St. Louis, MO, USA). Cells used for AFM measurements were plated onto 0.5% gelatin solution (G1393, Sigma, St. Louis, MO, USA)-coated coverslips in 35-mm Petri dishes. Cell-coated coverslips were placed in each dish with DMEM containing 2 mM L-glutamine, 1% antibiotic-antimycotic solution, 13.5 mM HEPES [pH 7.4], and 5% FBS to support mechanical testing. Adherent cells were cultivated in 25-cm^2^ flasks containing complete medium at 37 °C in a humidified atmosphere of 5% CO_2_.

### Cell irradiation

Cells were positioned in a chamber on irradiation equipment at the Heavy Ion Research Facility in Lanzhou (HIRFL). Cells were then exposed to ^12^C^6+^ions at an energy of 270 MeV/u and 13.3 keV/μm LET, using a dose rate of 0.3 Gy/min. The ^12^C^6+^ion dose of 0.5 Gy corresponded to a radiant fluence of approximately 2.35 × 10^7^ particles/cm^2^. The data were automatically collected using a microcomputer during irradiation, and the particle fluences of the beams were determined from an air ionization chamber signal based on the calibration of the detector (PTW-UNIDOS, Wiesbaden, Germany). Additionally, an approximate irradiation dose of 2 Gy at the 24 h time point was selected using the MTT assay (Fig. S2). All cells in the exponential growth phase were irradiated at a dose of 2 Gy at room temperature (RT), and the nonirradiated cells (0 Gy) were handled in parallel with the irradiated samples.

### Sodium dodecyl sulfate-polyacrylamide gel electrophoresis (SDS-PAGE) and IB analyses

Cells treated with CII for 0, 6, 12, or 24 h were evaluated by SDS-PAGE and IB analyses to assess protein expression. The cell lysates (50 mM Tris-HCl [pH 8.0]; 150 mM NaCl; NaN_3_; SDS 0.1%; NP-40 1%; and 100 μg/mL PMSF) were homogenized in solubilizing buffer supplemented with protease and phosphatase inhibitors (MSSAFE, Sigma, St. Louis, MO, USA). Subsequently, the cells were incubated on ice for 40 min. The supernatant was collected after centrifugation at 14,000 rpm for 20 min at 4 °C. The protein concentrations were quantified using the BCA method (23227, Pierce, Rockford, IL, USA). Prior to loading, the proteins were boiled in denaturation buffer (P0015L, Beyotime Institute of Biotechnology, Beijing, China) for 7 min at 100 °C. SDS-PAGE was performed using a discontinuous acrylamide gel^56^. Whole-cell lysate protein was loaded in each lane. A pre-stained molecular weight marker (SM1811, Fermentas, Burlington, ON, Canada) was used for size comparison. Each protein equivalent was separated by 10–12% SDS-PAGE and transferred onto PVDF membranes. The membranes were blocked in TBST buffer containing 5% nonfat dry milk (w/v) (D8340, BD Biosciences, Sparks, MD, USA) and then incubated overnight at 4 °C with primary antibodies against caspase-3 (ab17868), Bax (ab32124), and Bcl-2 (ab694) at the recommended dilutions (Abcam, San Francisco, CA, USA). The membranes were then incubated with horseradish peroxidase-conjugated goat anti-mouse (sc-2005)/anti-rabbit (sc-2004) secondary antibodies (Santa Cruz Biotechnology, Santa Cruz, CA, USA) at RT for 2 h. Next, they were developed using ECL reagent (6883, Cell Signaling, Danvers, MA, USA). The signals were visualized by exposure to the Chemi Doc™ XRS System with Image Lab™ software (Bio-Rad Laboratories, CA, USA). β-actin expression served as a control. Differences in protein expression were examined by densitometry analysis using ImageJ software (NIH, Maryland, USA, http://rsb.info.nih.gov/ij). IB experiments were repeated three times to confirm the results.

### Cluster-cell method for morphological analysis

The cluster-cell method (using ImageJ software) was adopted to measure the physical parameters of five HCC lines and one NH line in a field (×40). Cell borders were distinguished to achieve accurate cell size measurements per field^57^.

### Fluorescence imaging and F-actin cytoskeletal analysis

Cytoskeletal organization was detected based on the labeled F-actin in each cell line using IF imaging. The cells were grown on round coverslips at a density of 4,000 cells/cm^2^. The coverslips with plated cells were placed in a 6-well plate with 2.5 mL medium. After irradiation for 24 h, the cells were fixed for 10 min in 3.7% formaldehyde in phosphate-buffered saline (PBS; combined with 137 mM NaCl, 2.7 mM KCl, 10 mM Na_2_HPO_4_ and 1.8 mM KH_2_PO_4_, [pH 7.4]) at RT. Next, cells were permeabilized with 0.1% Triton X-100 in PBS for 3 min. F-actin was stained with a fluorescein isothiocyanate- phalloidin conjugate solution (P5282, Sigma, St. Louis, MO, USA). Then, 1% bovine serum albumin (BSA; 9048-46-8, Solarbio, Beijing, China) was added to the staining solution to reduce nonspecific background staining in these conjugates. The solution was placed on coverslips for 60 min in a dark room. After incubation, the coverslips were washed five times with PBS for 8 min each to remove unbound phalloidin conjugate. The cells on the coverslips were incubated with 4′, 6-diamidino-2-phenylindole (DAPI; D9542, Sigma, St. Louis, MO, USA) at 0.1 mg/mL in PBS for 15 min at RT and rinsed five times with PBS. The cells were sandwiched between the coverslip and a glass slide and mounted with antifade mounting medium (P0128, Beyotime, Beijing, China). Finally, the glass slides were examined using confocal microscopy (LSM700, Carl Zeiss, Jena, Germany). Multiple images of each cell line were captured to visualize the F-actin fibers and nuclear DNA. A typical image from each group is shown as a section of the cytoskeletal architecture. The mean intensities of F-actin in each cell were measured using ImageJ software.

### AFM for single-cell imaging and mechanical measurements

Topographical analysis and mechanical property measurements of individual cells were conducted using a biotype AFM. A JPK Nano Wizard III instrument (JPK Instruments AG, Germany) coupled with a Zeiss MicroImaging inverted optical microscope (Axiovert 200M, Carl Zeiss, Germany) was used with a 100 × 100 (fast × slow) μm^2^ scanner and closed-loop feedback system. A BioLever probe with a nominal spring constant of 0.03 N/m (BioLever- RC150VB-C1, Olympus Micro Cantilevers, Japan) was also used in this study. The cantilever tip was modified by attaching a smooth silicon dioxide microsphere with a diameter of 4.0 μm (EPR-Si-4, EPRUI Nanoparticles & Microspheres Co., Ltd., China) with epoxy resin glue (Epoxy F-05 Clear, Alteco, Japan) to simplify the contact geometry and minimize the lateral strain of the sample during indentation. The measurement procedures were conducted in medium at 37 °C using the BioCell liquid temperature-controlled chamber. The cells (at a density of 2 × 10^4^/mL) were seeded in advance on 24-mm sterile round gelatin-coated coverslips in plastic Petri dishes. All tests eliminated the confounding effects of neighboring cells on the edge contact and morphology to obtain accurate measurements of a single cell. Topography images and force spectroscopy measurements were conducted under contact mode with the medium. Detailed methods on force spectroscopy measurement are shown in Fig. S3.

### Statistical analysis

Data were presented as the mean ± standard deviation (SD). Difference in means was assessed by Student’s t-test and one-way ANOVA using the SPSS 17.0 software (IBM, Chicago, IL, USA). Force spectroscopy data were analyzed by using the Shapiro–Wilk test. Associations among cell morphology, mechanical properties, actin cytoskeleton architecture, and apoptotic protein expression were tested by using Pearson’s product–moment correlation coefficient. Statistical significance was set at *P* < 0.05.

## Additional Information

**How to cite this article**: Zhang, B. *et al*. Carbon Ion-Irradiated Hepatoma Cells Exhibit Coupling Interplay between Apoptotic Signaling and Morphological and Mechanical Remodeling. *Sci. Rep.* **6**, 35131; doi: 10.1038/srep35131 (2016).

## Supplementary Material

Supplementary Information

## Figures and Tables

**Table 1:** Changes in the geometrical parameters of HCCs and NHs before and after CII.

Method	Group	Cell objects	Length (μm)	Width (μm)	Perimeter (μm)	Thickness (μm)	Cell projected area (μm^2^)	Volume (μm^3^)
Cluster cell analysis	L02-0Gy	1788	65.46 ± 16.45	39.39 ± 13.59	44.13 ± 13.89	6.37 ± 1.05	522.73 ± 171.43	1168.84 ± 569.42
	HepG2-0Gy	1495	62.51 ± 16.96	36.53 ± 10.15	48.34 ± 14.12	6.23 ± 0.85	587.30 ± 155.97	1447.10 ± 575.99
	Smmc-7221-0Gy	1095	67.57 ± 15.12	33.10 ± 11.02	50.19 ± 15.58	6.72 ± 1.14	584.06 ± 194.55	1382.64 ± 674.20
	Huh-7-0Gy	1025	61.48 ± 17.73	32.42 ± 11.17	53.67 ± 18.09	6.86 ± 1.11	606.97 ± 192.12	1459.43 ± 672.20
	Mhcc-97L-0Gy	1883	55.75 ± 18.31	36.47 ± 12.50	54.72 ± 14.28	7.15 ± 1.08	656.95 ± 192.07	1634.82 ± 694.35
	Mhcc-97H-0Gy	1285	59.47 ± 17.54	34.84 ± 9.46	52.70 ± 14.64	7.19 ± 0.86	611.43 ± 141.70	1399.93 ± 474.39
	L02 + 2Gy	308	41.50 ± 16.07*	30.84 ± 10.75*	29.80 ± 7.30*	4.41 ± 0.63*	307.50 ± 77.36*	519.22 ± 192.32*
	HepG2 + 2Gy	445	37.11 ± 14.47*	26.05 ± 9.16*	31.36 ± 8.23*	4.95 ± 0.62*	313.57 ± 73.60*	531.57 ± 186.43*
	Smmc-7221 + 2Gy	297	39.13 ± 13.62*	27.66 ± 11.72*	30.08 ± 7.20*	5.14 ± 0.45*	333.94 ± 57.07*	580.31 ± 145.12*
	Huh-7 + 2Gy	308	41.10 ± 11.01*	29.11 ± 7.57*	32.05 ± 6.85*	5.39 ± 0.56*	368.78 ± 74.91*	676.40 ± 203.21*
	Mhcc-97L + 2Gy	260	47.83 ± 13.34*	32.37 ± 7.39*	36.70 ± 10.83*	5.64 ± 0.73*	405.96 ± 101.03*	787.33 ± 284.24*
	Mhcc-97H + 2Gy	420	52.16 ± 10.81*	33.59 ± 6.81†	45.67 ± 8.01*	6.01 ± 0.42*	455.42 ± 62.03*	920.51 ± 185.02*
Single cell analysis	L02-0Gy	25	88.87 ± 6.92	56.49 ± 6.47	41.69 ± 5.02	6.62 ± 0.44	555.97 ± 95.63	1240.70 ± 246.23
	HepG2-0Gy	21	77.60 ± 6.30	53.07 ± 6.34	47.06 ± 4.85	7.49 ± 0.77	712.52 ± 141.38	1347.78 ± 302.84
	Smmc-7221-0Gy	39	89.34 ± 6.93	47.50 ± 8.80	39.62 ± 8.15	6.31 ± 1.30	520.53 ± 181.50	1184.84 ± 341.77
	Huh-7-0Gy	21	83.12 ± 5.26	52.17 ± 9.34	41.29 ± 3.05	6.58 ± 0.49	545.88 ± 80.13	1208.83 ± 264.63
	Mhcc-97L-0Gy	29	71.67 ± 9.65	48.79 ± 9.71	42.65 ± 5.21	6.79 ± 0.83	587.56 ± 133.79	1368.19 ± 381.10
	Mhcc-97H-0Gy	22	70.75 ± 6.04	45.63 ± 7.33	43.48 ± 2.79	6.92 ± 0.61	603.20 ± 90.84	1396.96 ± 174.03
	L02 + 2Gy	29	39.85 ± 4.02**	30.19 ± 3.37**	27.53 ± 3.17**	4.38 ± 0.51**	244.42 ± 55.75**	366.44 ± 98.29**
	HepG2 + 2Gy	20	36.30 ± 7.97**	26.82 ± 8.94**	28.07 ± 3.09**	4.55 ± 0.39**	251.58 ± 45.93**	396.59 ± 58.04**
	Smmc-7221 + 2Gy	30	42.05 ± 8.52**	31.32 ± 7.93**	30.67 ± 4.25**	4.88 ± 0.55**	301.07 ± 56.42**	494.85 ± 92.57**
	Huh-7 + 2Gy	20	45.24 ± 3.65**	33.35 ± 4.81**	32.27 ± 5.29**	5.12 ± 0.84**	329.55 ± 69.81**	564.53 ± 113.87**
	Mhcc-97L + 2Gy	31	58.96 ± 7.62**	43.42 ± 5.91**	34.15 ± 4.37**	5.44 ± 0.70**	377.25 ± 61.10**	704.82 ± 142.24**
	Mhcc-97H + 2Gy	24	63.85 ± 6.89**	44.01 ± 6.49†	39.18 ± 3.34**	6.24 ± 0.53**	492.16 ± 84.97**	809.29 ± 263.51**

Note. Values are expressed as the mean ± SD. *Cluster-cell analysis, *P* < 0.05. **Single-cell analysis, *P* < 0.05. **†**Single-cell and cluster-cell analysis, *P* > 0.05. The analyses were performed using Student’s t-test for independent samples.

## References

[b1] Frese M. C., Victor K. Y., Stewart R. D. & Carlson D. J. A mechanism-based approach to predict the relative biological effectiveness of protons and carbon ions in radiation therapy. Int. J. Radiat. Oncol. 83, 442–450 (2012). 10.1016/j.ijrobp.2011.06.1983 22099045

[b2] Suzuki M., Kase Y., Yamaguchi H., Kanai T. & Ando K. Relative biological effectiveness for cell-killing effect on various human cell lines irradiated with heavy-ion medical accelerator in Chiba (HIMAC) carbon ion beams. Int. J. Radiat. Oncol. 48, 241–250 (2000). 10.1016/s0360-3016(00)00568-x 10924995

[b3] Zhang H. . Results of carbon ion radiotherapy for skin carcinomas in 45 patients. Br. J. Dermatol. 166, 1100–1106 (2012). 22136631 10.1111/j.1365-2133.2011.10764.x

[b4] Durante M. & Loeffler J. S. Charged particles in radiation oncology. Nat. Rev. Clin. Oncol. 7, 37–43 (2010). 19949433 10.1038/nrclinonc.2009.183

[b5] Suit H. . Proton vs carbon ion beams in the definitive radiation treatment of cancer patients. Radiother. Oncol. 95, 3–22 (2010). 20185186 10.1016/j.radonc.2010.01.015

[b6] Jinno-Oue A. . Irradiation with carbon ion beams induces apoptosis, autophagy, and cellular senescence in a human glioma-derived cell line. Int. J. Radiat. Oncol. 76, 229–241 (2010). 10.1016/j.ijrobp.2009.08.054 20005456

[b7] Kerr J. F., Wyllie A. H. & Currie A. R. Apoptosis: a basic biological phenomenon with wide-ranging implications in tissue kinetics. Br. J. Cancer 26, 239–257 (1972). 4561027 10.1038/bjc.1972.33 PMC2008650

[b8] Marino G., Niso-Santano M., Baehrecke E. H. & Kroemer G. Self-consumption: the interplay of autophagy and apoptosis. Nat. Rev. Mol. Cell Biol. 15, 81–94 (2014). 24401948 10.1038/nrm3735 PMC3970201

[b9] Elmore S. Apoptosis: a review of programmed cell death. Toxicol. Pathol. 35, 495–516 (2007). 17562483 10.1080/01926230701320337 PMC2117903

[b10] Fuchs Y. & Steller H. Programmed cell death in animal development and disease. Cell 147, 742–758 (2011). 22078876 10.1016/j.cell.2011.10.033 PMC4511103

[b11] Ziegler U. & Groscurth P. Morphological features of cell death. Physiology 19, 124–128 (2004). 10.1152/nips.01519.2004 15143207

[b12] Fulda S. Molecular pathways: targeting inhibitor of apoptosis proteins in cancer-from molecular mechanism to therapeutic application. Clin. Cancer Res. 20, 289–295 (2014). 24270683 10.1158/1078-0432.CCR-13-0227

[b13] Czabotar P. E., Lessene G., Strasser A. & Adams J. M. Control of apoptosis by the Bcl-2 protein family: implications for physiology and therapy. Nat. Rev. Mol. Cell Biol. 15, 49–63 (2014). 24355989 10.1038/nrm3722

[b14] Lecuit T. & Lenne P. F. Cell surface mechanics and the control of cell shape, tissue patterns and morphogenesis. Nat. Rev. Mol. Cell Biol. 8, 633–644 (2007). 17643125 10.1038/nrm2222

[b15] DuFort C. C., Paszek M. J. & Weaver V. M. Balancing forces: architectural control of mechanotransduction. Nat. Rev. Mol. Cell Biol. 12, 308–319 (2011). 21508987 10.1038/nrm3112 PMC3564968

[b16] Hoffman B. D., Grashoff C. & Schwartz M. A. Dynamic molecular processes mediate cellular mechanotransduction. Nature 475, 316–323 (2011). 21776077 10.1038/nature10316 PMC6449687

[b17] Bakal C., Aach J., Church G. & Perrimon N. Quantitative morphological signatures define local signaling networks regulating cell morphology. Science 316, 1753–1756 (2007). 17588932 10.1126/science.1140324

[b18] Wada K. I., Itoga K., Okano T., Yonemura S. & Sasaki H. Hippo pathway regulation by cell morphology and stress fibers. Development 138, 3907–3914 (2011). 21831922 10.1242/dev.070987

[b19] Cross S. E., Jin Y. S., Rao J. & Gimzewski J. K. Nanomechanical analysis of cells from cancer patients. Nat. Nanotechnol. 2, 780–783 (2007). 18654431 10.1038/nnano.2007.388

[b20] Xu W. . Cell stiffness is a biomarker of the metastatic potential of ovarian cancer cells. PloS One 7, e46609 (2012). 23056368 10.1371/journal.pone.0046609 PMC3464294

[b21] Swaminathan V. . Mechanical stiffness grades metastatic potential in patient tumor cells and in cancer cell lines. Cancer Res. 71, 5075–5080 (2011). 21642375 10.1158/0008-5472.CAN-11-0247 PMC3220953

[b22] Sokolov I., Dokukin M. E. & Guz N. V. Method for quantitative measurements of the elastic modulus of biological cells in AFM indentation experiments. Methods 60, 202–213 (2013). 23639869 10.1016/j.ymeth.2013.03.037

[b23] Dufrêne Y. F., Martínez-Martín D., Medalsy I., Alsteens D. & Müller D. J. Multiparametric imaging of biological systems by force-distance curve-based AFM. Nat. Methods 10, 847–854 (2013). 23985731 10.1038/nmeth.2602

[b24] Hengartner M. O. The biochemistry of apoptosis. Nature 407, 770–776 (2000). 11048727 10.1038/35037710

[b25] Earnshaw W. C., Martins L. M. & Kaufmann S. H. Mammalian caspases: structure, activation, substrates, and functions during apoptosis. Annu. Rev. Biochem. 68, 383–424 (1999). 10872455 10.1146/annurev.biochem.68.1.383

[b26] Thornberry N. A. & Lazebnik Y. Caspases: enemies within. Science 281, 1312–1316 (1998). 9721091 10.1126/science.281.5381.1312

[b27] Tomiyama A. . MEK–ERK-dependent multiple caspase activation by mitochondrial proapoptotic Bcl-2 family proteins is essential for heavy ion irradiation-induced glioma cell death. Cell Death Dis. 1, e60 (2010). 21364665 10.1038/cddis.2010.37 PMC3039836

[b28] Takahashi A. . High-LET radiation enhanced apoptosis but not necrosis regardless of p53 status. Int. J. Radiat. Oncol. 60, 591–597 (2004). 10.1016/j.ijrobp.2004.05.062 15380596

[b29] Ye F. . Long-term autophagy and Nrf2 signaling in the hippocampi of developing mice after carbon ion exposure. Sci. Rep. 5 (2015). 10.1038/srep18636 PMC4686898 26689155

[b30] Sun C. . Carbon ion beams induce hepatoma cell death by NADPH oxidase-mediated mitochondrial damage. J. Cell Physiol. 229, 100–107 (2014). 23804302 10.1002/jcp.24424

[b31] Shi Y. Mechanisms of caspase activation and inhibition during apoptosis. Mol. Cell 9, 459–470 (2002). 11931755 10.1016/s1097-2765(02)00482-3

[b32] McIlwain D. R., Berger T. & Mak T. W. Caspase functions in cell death and disease. Cold Spring Harb Perspect Biol. 5, a008656 (2013). 23545416 10.1101/cshperspect.a008656 PMC3683896

[b33] Slee E. A. . Ordering the cytochrome c-initiated Caspase Cascade: hierarchical activation of caspases-2,-3,-6,-7,-8, and-10 in a caspase-9-dependent manner. J. Cell Biol. 144, 281–292 (1999). 9922454 10.1083/jcb.144.2.281 PMC2132895

[b34] Porter A. G. & Jänicke R. U. Emerging roles of caspase-3 in apoptosis. Cell Death Differ. 6, 99–104 (1999). 10200555 10.1038/sj.cdd.4400476

[b35] Chipuk J., Bouchier-Hayes L. & Green D. Mitochondrial outer membrane permeabilization during apoptosis: the innocent bystander scenario. Cell Death Differ. 13, 1396–1402 (2006). 16710362 10.1038/sj.cdd.4401963

[b36] Leri A. . Stretch-mediated release of angiotensin II induces myocyte apoptosis by activating p53 that enhances the local renin-angiotensin system and decreases the Bcl-2-to-Bax protein ratio in the cell. J. Clin. Invest. 101, 1326 (1998). 9525975 10.1172/JCI316 PMC508710

[b37] Janmey P. A. The cytoskeleton and cell signaling: component localization and mechanical coupling. Physiol. Rev. 78, 763–781 (1998). 9674694 10.1152/physrev.1998.78.3.763

[b38] Celik E., Abdulreda M. H., Maiguel D., Li J. & Moy V. T. Rearrangement of microtubule network under biochemical and mechanical stimulations. Methods 60, 195–201 (2013). 23466787 10.1016/j.ymeth.2013.02.014 PMC3669668

[b39] Stewart M. P., Toyoda Y., Hyman A. A. & Muller D. J. Tracking mechanics and volume of globular cells with atomic force microscopy using a constant-height clamp. Nat. Protoc. 7, 143–154 (2012). 22222789 10.1038/nprot.2011.434

[b40] Kulms D. . Apoptosis induced by disruption of the actin cytoskeleton is mediated via activation of CD95 (Fas/APO-1). Cell Death Differ. 9, 598–608 (2002). 12032668 10.1038/sj.cdd.4401002

[b41] Gourlay C. W. & Ayscough K. R. The actin cytoskeleton: a key regulator of apoptosis and ageing? Nat. Rev. Mol. Cell Biol. 6, 583–589 (2005). 16072039 10.1038/nrm1682

[b42] Shah E. A. & Keren K. Mechanical forces and feedbacks in cell motility. Curr. Opin. Cell Biol. 25, 550–557 (2013). 23860439 10.1016/j.ceb.2013.06.009

[b43] Cabado A., Leira F., Vieytes M., Vieites J. & Botana L. Cytoskeletal disruption is the key factor that triggers apoptosis in okadaic acid-treated neuroblastoma cells. Arch. Toxicol. 78, 74–85 (2004). 14652712 10.1007/s00204-003-0505-4

[b44] Kothakota S. . Caspase-3-generated fragment of gelsolin: effector of morphological change in apoptosis. Science 278, 294–298 (1997). 9323209 10.1126/science.278.5336.294

[b45] Sun H. Q., Yamamoto M., Mejillano M. & Yin H. L. Gelsolin, a multifunctional actin regulatory protein. J. Biol. Chem. 274, 33179–33182 (1999). 10559185 10.1074/jbc.274.47.33179

[b46] Yamazaki Y. . Cytoskeletal disruption accelerates caspase-3 activation and alters the intracellular membrane reorganization in DNA damage-induced apoptosis. Exp. Cell Res. 259, 64–78 (2000). 10942579 10.1006/excr.2000.4970

[b47] Suria H., Chau L. A., Negrou E., Kelvin D. J. & Madrenas J. Cytoskeletal disruption induces T cell apoptosis by a caspase-3 mediated mechanism. Life Sci. 65, 2697–2707 (1999). 10622279 10.1016/s0024-3205(99)00538-x

[b48] Geng Y. J. . Caspase-3-induced gelsolin fragmentation contributes to actin cytoskeletal collapse, nucleolysis, and apoptosis of vascular smooth muscle cells exposed to proinflammatory cytokines. Eur. J. Cell Biol. 77, 294–302 (1998). 9930654 10.1016/S0171-9335(98)80088-5

[b49] Saraste A. & Pulkki K. Morphologic and biochemical hallmarks of apoptosis. Cardiovasc. Res. 45, 528–537 (2000). 10728374 10.1016/s0008-6363(99)00384-3

[b50] Van Cruchten S. & Van Den Broeck W. Morphological and biochemical aspects of apoptosis, oncosis and necrosis. Anat. Histol. Embryol. 31, 214–223 (2002). 12196263 10.1046/j.1439-0264.2002.00398.x

[b51] Kerr J. F., Winterford C. M. & Harmon B. V. Apoptosis. Its significance in cancer and cancer therapy. Cancer 73, 2013–2026 (1994). 8156506 10.1002/1097-0142(19940415)73:8<2013::aid-cncr2820730802>3.0.co;2-j

[b52] Dinsmore A. D., Yodh A. G. & Pine D. J. Phase diagrams of nearly-hard-sphere binary colloids. Phys. Rev. E 52, 4045–4057 (1995). 10.1103/physreve.52.4045 9963877

[b53] Imhof A. & Dhont J. K. Experimental phase diagram of a binary colloidal hard-sphere mixture with a large size ratio. Phys. Rev. Lett. 75, 1662–1665 (1995). 10060354 10.1103/PhysRevLett.75.1662

[b54] Steiner U., Meller A. & Stavans J. Entropy driven phase separation in binary emulsions. Phys. Rev. Lett. 74, 4750–4753 (1995). 10058589 10.1103/PhysRevLett.74.4750

[b55] Ilett S. M., Orrock A., Poon W. C. & Pusey P. N. Phase behavior of a model colloid-polymer mixture. Phys. Rev. E 51, 1344–1352 (1995). 10.1103/physreve.51.1344 9962777

[b56] Makarova G. . Epidermal growth factor-induced modulation of cytokeratin expression levels influences the morphological phenotype of head and neck squamous cell carcinoma cells. Cell Tissue Res. 351, 59–72 (2013). 23111772 10.1007/s00441-012-1500-y

[b57] Zhou T., Marx K. A., Dewilde A. H., McIntosh D. & Braunhut S. J. Dynamic cell adhesion and viscoelastic signatures distinguish normal from malignant human mammary cells using quartz crystal microbalance. Anal. Biochem. 421, 164–171 (2012). 22119070 10.1016/j.ab.2011.10.052

