# Supplementary material for: Carbon Ion-Irradiated Hepatoma Cells Exhibit Coupling Interplay between Apoptotic Signaling and Morphological and Mechanical Remodeling
Source: Sci Rep. 2016 Oct 12;6:35131. doi: 10.1038/srep35131 (PMC5059721; doi:10.1038/srep35131)
Supplement: Supplementary Information [file srep35131-s1.doc]

**SUPPLEMENTARY INFORMATION**

**Carbon Ion-Irradiated Hepatoma Cells Exhibit Coupling Interplay between Apoptotic Signaling and Morphological and Mechanical Remodeling**

**Baoping Zhang^1,2,3^, Long Li^1,3^,** **Zhiqiang Li^4^, Yang Liu^2,†^, Hong Zhang^2^****, and Jizeng Wang^1,3,†^**

***^1^****Key Laboratory of Mechanics on Disaster and Environment in Western China, Ministry of Education, College of Civil Engineering and Mechanics, Lanzhou University, Lanzhou,* *730000, China.*

***^2^****Department of Heavy Ion Radiation Medicine, Institute of Modern Physics, Chinese Academy of Sciences, Lanzhou, 730000, China.*

***^3^****Institute of Biomechanics and Medical Engineering, Lanzhou University, Lanzhou, 730000, China.*

***^4^****Key Laboratory of Oral Diseases of Gansu Province, Northwest University for Nationalities, Lanzhou, 730030, China.*

**^†^Corresponding Author**

Jizeng Wang

E-mail: [jzwangibme@gmail.com](mailto:jzwangibme@gmail.com )

Yang Liu

E-mail: [liuy@impcas.ac.cn](mailto:liuy@impcas.ac.cn)

**Supplementary Discussion**

Cellular Budding of Apoptotic Bodies (ABs)

As shown in Fig. S1A, we considered an ideal theoretical model to understand the budding behavior of an AB quantitatively. In this model, the cell was modeled as a spherical vesicle with radius *R*_c_, inside of which were small molecules with effective radius *R*_s_ and number density $\rho_{\text{s}}$ and the AB with effective radius *R*_b_. The membrane has bending and tension moduli of *κ* and *γ*. Typically, $\kappa=20\text{ }k_{\text{B}}T$[^1^](#_ENREF_1) and $\gamma\approx0.005\text{ }k_{\text{B}}T/\text{nm}^{2}$, [^2^](#_ENREF_2) where $k_{\text{B}}$ and *T* are the Boltzmann constant and absolute temperature, respectively.

Upon addition of numerous smaller disassembled molecules, density gradients lead to an effective force field that pushes the larger AB toward the cell membrane during CA. The AB can eventually be driven to separate from the apoptotic cell because of the depletion interactions among molecules to maximize the mixing entropy. The depletion force exerted by the cytoplasm on the AB can be expressed as follows:[^3^](#_ENREF_3)

$F_{\text{d}}=4\pi k_{\text{B}}T\rho_{\text{s}}\frac{R_{\text{c}}R_{\text{b}}R_{\text{s}}\left(R_{\text{c}}-R_{\text{b}}-R_{\text{s}}\right)}{\left(R_{\text{c}}-R_{\text{b}}\right)^{2}}.$ (S1.1)

This force pushes the AB against the cell membrane, causing the membrane to wrap around the AB locally at the cost of elevated elastic energy associated with increased local curvature of the membrane, as shown in [Fig. S1A](http://www.pnas.org/content/102/27/9469.full#F2). As the cell can be much larger than the AB, we assumed a 3D configuration of an infinite flat membrane wrapping around a spherical particle. We adopted the mathematical framework developed in the study on how a cell membrane wraps around a spherical particle. Thus, the deformation energy of the cell membrane can be written as follows:

$E_{\text{m}}\left(h\right)=\int\left(2\kappa H^{2}+\gamma\right)\text{d}A$ (S1.2)

where *H* = 1/*R*_b_ is the mean curvature, *h* is the budding depth shown in Fig. S1A, and *A* = 2*πR*_b_*h* is the contact area between the AB and membrane. By considering the geometrical relationship shown in Fig. S1A, Equation (S1.2) can be further expressed as follows:

$\frac{E_{m}\left(h\right)}{4\pi R_{\text{b}}^{2}}\approx\frac{\kappa h}{R_{\text{b}}^{3}}+\gamma\left(\frac{h}{2R_{\text{b}}}-\frac{2R_{\text{b}}h-h^{2}}{4R_{\text{b}}^{2}}\right).$ (S1.3)

For complete budding of the AB or *h* = 2*R*_b_, we now assumed that the repulsive barrier needs to be overcome. Thus, the net driving force can be expressed as follows:

$F=F_{\text{d}}-\frac{\partial E_{\text{m}}}{\partial h}|_{h=2R_{\text{b}}}=F_{\text{d}}-4\pi\left(\frac{\kappa}{R_{\text{b}}}+\gamma R_{\text{b}}\right).$ (S1.4)

A typical cancer cell contains several billions of molecules[^6^](#_ENREF_6). Assuming that *R*_c_ = 10 µm and that the total number of molecules is *N* = 5 × 10^9^, estimation of the bulk number density of the completely disassembled molecules is $\rho_{\text{s}}\approx N/(4\pi R_{\text{c}}^{3}/3)=0.001\text{ nm}^{-3}$, which is of the same order of magnitude as that in[^7^](#_ENREF_7). As observed in Fig. 5B, the AB radius ranges from 0.2 µm to 2 µm, which corresponds to typical organelle sizes. For the effective hydrodynamic radius of the small disassembled molecules, the F-actin fragments can be selected as the representative example. These fragments have a diameter of *d* = 8 nm and a typical length of *L* = 150 nm [^10^](#_ENREF_10). Their effective hydrodynamic radius can be estimated as follows:

$R_{\text{s}}\sim L\left(\frac{3\alpha^{2}}{16}\right)^{1/3}\left[1.009+1.395\times10^{-2}\ln\alpha+7.880\times10^{-2}\ln\alpha^{2}\right.+\left.6.04\times10^{-3}\ln\alpha^{3}\right]\approx19 \text{nm}$ (S1.5)

where *α*=*L*/*d*.

If we assume that a successful budding event corresponds to a positive net driving force, as shown in Equation (S1.4), we can create a phase diagram (Fig. S1B) to illustrate how the combination of number density $\rho_{\text{s}}$ and radius of the AB *R*_b_ can ensure effective budding. As shown in Fig. S1B, the number density of small disassembled molecules $\rho_{s}\sim0.001 \text{nm}^{-3}$ can ensure effective budding of ABs with sizes from hundreds of nanometers to microns.

**Supplementary Method**

AFM Single-Cell Topography and Mechanical Testing

Topographical Imaging

Topographical imaging was conducted on a detachable substrate with a round coverslip that was mounted on a BioCell temperature-controlled chamber (JPK Instruments AG, Germany). The cultivated cells were briefly replaced with high-glucose Dulbecco’s modified Eagle’s medium supplemented with 5% (v/v) FBS containing 13.5 mM HEPES (pH 7.2) as the culture media during force spectroscopy measurement. The cells were scanned by using a scale of 50 × 50 µm^2^ and a pixel resolution of 512 × 512 at a line rate of 0.10–0.20 Hz, with a *z*-piezo displacement between 5 µm and 8 µm to obtain better imaging. For each cell line, approximately 30 individual cells in each group were randomly selected for indentation, and 6 measurement points on the surface of each cell were examined. Each point was measured 10 times. The force curves were collected on the glass substrate to calibrate the deflection sensitivity of the instrument before indenting the sample.

Cell Height and Surface Roughness

The topographical image was examined for cell height measurement through cross-sectional analysis using the JPK data processing software (http://www.jpk.com). Surface roughness analysis was conducted in terms of arithmetic average (*R*a) and root mean square (*R*q) heights[^13^](#_ENREF_13), and scanning windows of 10 × 10 µm^2^ local areas were selected. Statistical analysis using a two-sample independent Student’s *t* test was applied to all pairs of samples (irradiated and nonirradiated cells) to determine the significant differences in *R*a and *R*q, where *P* *<* 0.05 was considered significant.

Elastic Modulus, Adhesion Force, and Surface Adhesion Energy

Spherically tipped cantilevers (4 µm diameter, *k* ∼ 0.03 N/m, BioLever-RC150VB-C1, Olympus Micro Cantilevers) were briefly used in the indentation experiments. The rounded tip can minimize the damage to the cell membrane during contact and reduce the nonlinear effect of cell deformation because of a more homogenous contact between the cells and the probe[^14-18^](#_ENREF_14). Individual cells were mechanically tested using single indentation test to distinguish the nuclear and cytoplasmic regions of the cell (Fig. S3). Force–distance curves were acquired at a sample rate of 5 kHz and a constant approaching and retracting velocity of ∼1 µm/s. A surface delay of 1 s was selected to guarantee that time is sufficient for the interaction between the cell surface and tip and stress relaxation at the initial drop[^21^](#_ENREF_21). The preset force was fixed to 1.0 ± 0.5 nN for all force curves so that all tests are comparable. The maximum indentation was kept less than 10% of the total cell thickness to avoid the influence of the substrate on the measurements[^22^](#_ENREF_22).

For Young’s modulus of the cell, *E*_elastic_ was extracted from the force curves based on the Hertz contact model as follows:

$F(\delta)^{2/3}=\left[\frac{4\sqrt{R}}{3(1-\nu^{2})}E_{elastic}\right]^{2/3}\delta$ (S3.1)

where *F* is the applied loading force, *R* is the relative radius of the tip, and *v* is the Poisson ratio, *δ* = (*z* − *z*_0_) − (*d* − *d*_0_) represents the difference in the relative changes of the piezo stack movement (*z*) and cantilever deflection (*d*), and *z*_0_ and *d*_0_ are the values of *z* and *d* at the contact point, respectively. In this study, *v* = 0.5 was selected by assuming that the cell as a biomaterial is incompressible[^26-28^](#_ENREF_26).

The adhesion force and surface adhesion energy were determined during retraction of the cantilever at a prescribed speed. As shown in the figure inset of Fig. S3C, we recorded the magnitude of the maximum detachment force as the adhesion or pull-off force. The surface adhesion energy was actually the so-called work of adhesion obtained by calculating the shaded area in the retraction force curve, as shown in the figure inset of Fig. S3C. All these processes were completed by using the JPK data processing software (http://www.jpk.com) with two proprietary codes, namely, a topographical image analysis code and a force spectroscopy curve analysis code.

**Supplementary Figures**

**
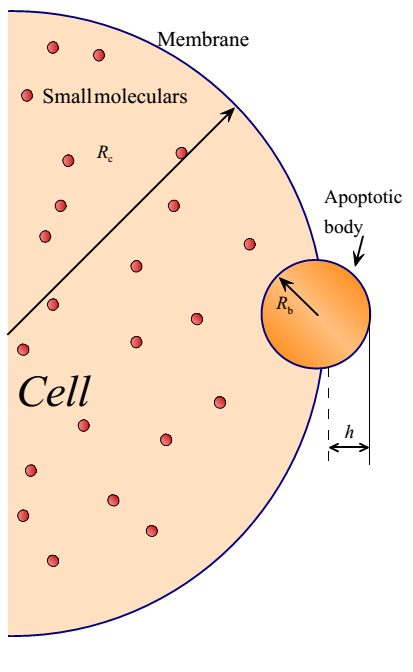
**

(A)


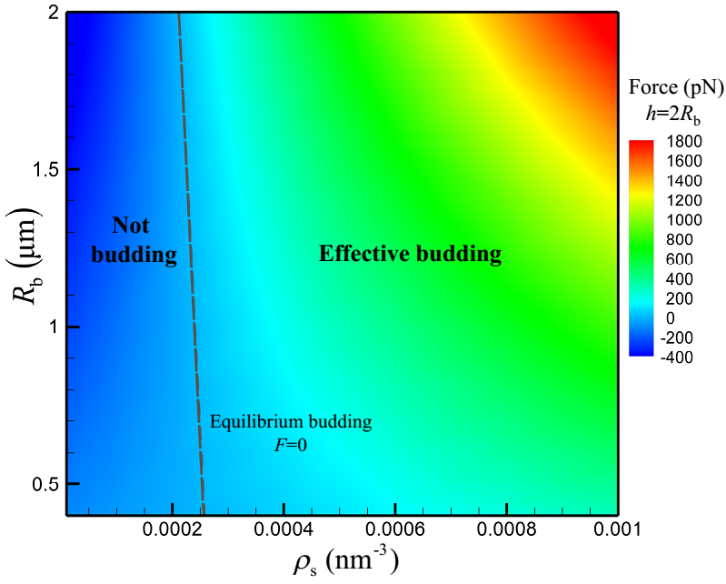


(B)

Figure S1. Cellular budding of apoptotic bodies (ABs). (A) Schematic plot of AB budding from a cell. (B) Phase diagram of AB budding from a cell.


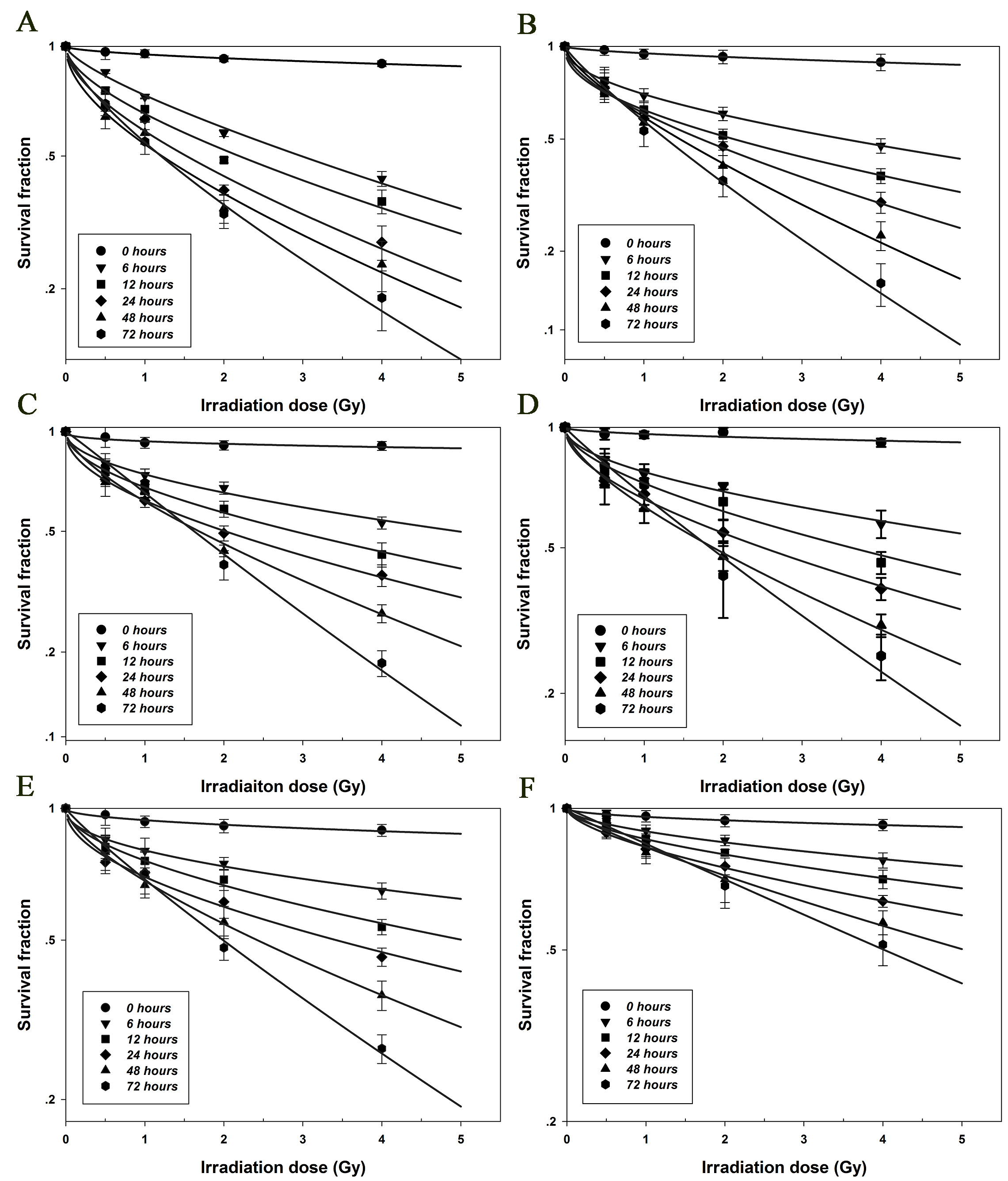


Figure S2. Survival curves for human HCC lines within 72 h induced by CII. (A) NH L02; (B) HCC HepG2. (C) HCC Huh-7. (D) HCC Smmc-7721. (E) HCC Mhcc-97L and (F) HCC Mhcc-97H. The mean ± SD is shown in terms of three different experiments. Statistical methods are Student’s t-test and one-way ANOVA, *P* < 0.05.


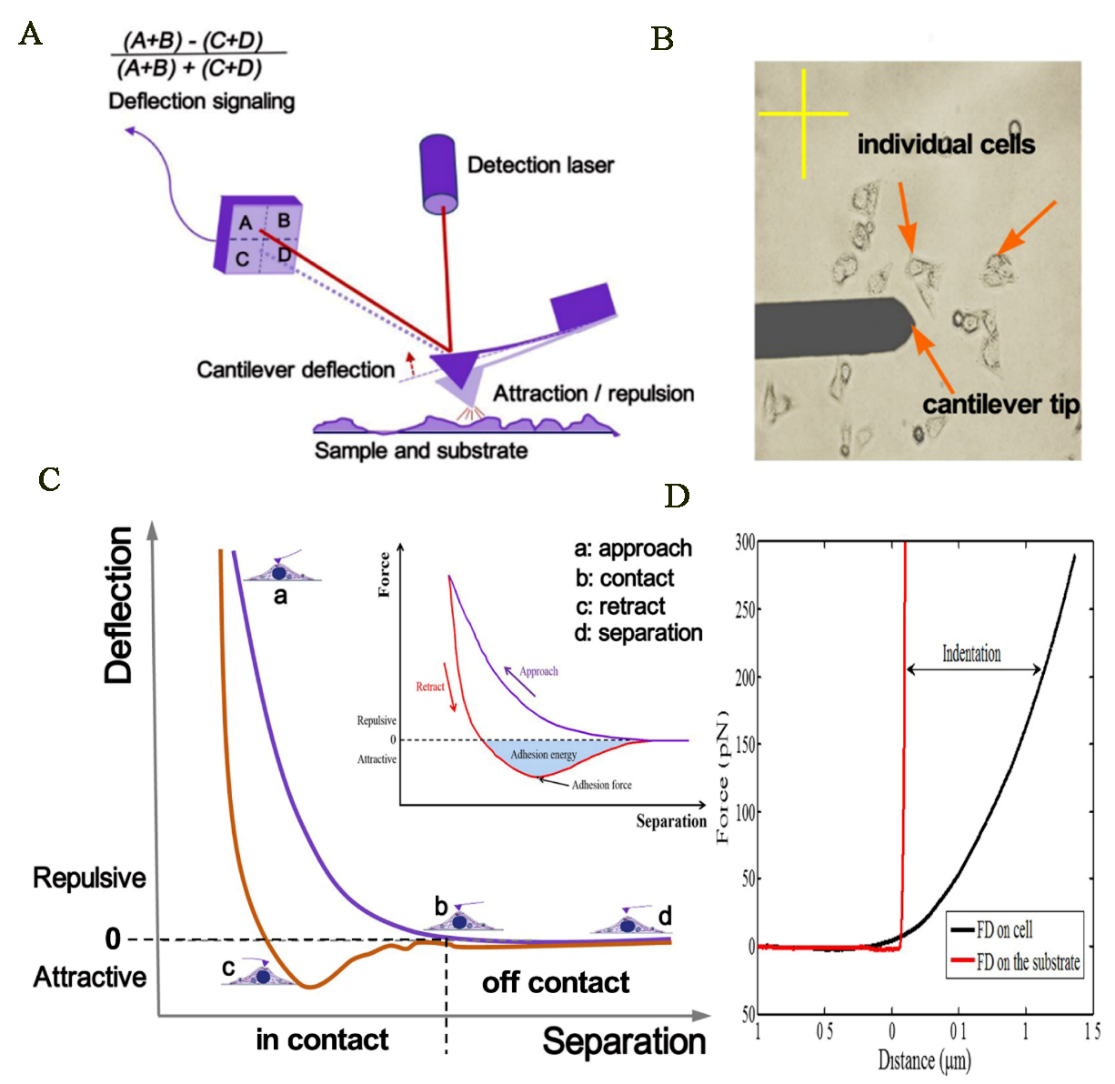


Figure S3. Schematic of the basic principle of biotype AFM. (A) AFM with a microscale cantilever and a specifically shaped tip is used to scan the cell surface. (B) Nanoscale imaging platform combining advanced AFM capabilities with modern optical microscopy. (C) AFM force–distance cycle for approach (blue) and retraction (red) from the surface, and (D) A typical force–distance curve in Young’s modulus determination.

**Bibliographic references**

1. Evans, E. & Rawicz, W. Entropy-driven tension and bending elasticity in condensed-fluid membranes. *Phys. Rev. Lett.* **64**, 2094 (1990).

2. Li, L., Liu, X., Zhou, Y. & Wang, J. On resistance to virus entry into host cells. *Biophys. J.* **102**, 2230-2233 (2012).

3. Li, W. & Ma, H. Depletion potential near curved surfaces. *Phys. Rev. E* **66**, 061407 (2002).

4. Gao, H., Shi, W. & Freund, L. B. Mechanics of receptor-mediated endocytosis. *Proc. Natl. Acad. Sci. USA.* **102**, 9469-9474 (2005).

5. Wang, J. & Li, L. Coupled elasticity-diffusion model for the effects of cytoskeleton deformation on cellular uptake of cylindrical nanoparticles. *J. R. Soc. Interface* **12**, 20141023 (2015).

6. Alberts, B. *et al.* Molecular Biology of the Cell. 4nd edn. Garland Science, New York (2002).

7. Liu, Y. *et al.* Depletion effect and biomembrane budding. *J. Biol. Phys.* **39**, 665-671 (2013).

8. Fowler, W. E. & Aebi, U. A consistent picture of the actin filament related to the orientation of the actin molecule. *J. Cell Biol.* **97**, 264-269 (1983).

9. Xu, K., Babcock, H. P. & Zhuang, X. Dual-objective STORM reveals three-dimensional filament organization in the actin cytoskeleton. *Nat. Methods* **9**, 185-188 (2012).

10. Shen, B. W., Josephs, R. & Steck, T. L. Ultrastructure of the intact skeleton of the human erythrocyte membrane. *J. Cell Biol.* **102**, 997-1006 (1986).

11. Shi, W., Wang, J., Fan, X. & Gao, H. Size and shape effects on diffusion and absorption of colloidal particles near a partially absorbing sphere: implications for uptake of nanoparticles in animal cells. *Phys. Rev. E* **78**, 061914 (2008).

12. Ortega, A. & de la Torre, J. G. Hydrodynamic properties of rodlike and disklike particles in dilute solution. *J. Chem. Phys.* **119**, 9914-9919 (2003).

13. Kim, K. S. *et al.* AFM-detected apoptotic changes in morphology and biophysical property caused by paclitaxel in Ishikawa and HeLa cells. *PLoS One* **7**, e30066 (2012).

14. Ketene, A. N., Roberts, P. C., Shea, A. A., Schmelz, E. M. & Agah, M. Actin filaments play a primary role for structural integrity and viscoelastic response in cells. *Integr. Biol.* **4**, 540-549 (2012).

15. González-Cruz, R. D., Fonseca, V. C. & Darling, E. M. Cellular mechanical properties reflect the differentiation potential of adipose-derived mesenchymal stem cells. *Proc. Natl. Acad. Sci. USA.* **109**, 1523-1529 (2012).

16. Ketene, A. N., Schmelz, E. M., Roberts, P. C. & Agah, M. The effects of cancer progression on the viscoelasticity of ovarian cell cytoskeleton structures. *Nanomed. Nanotechnol.* **8**, 93-102 (2012).

17. Darling, E., Zauscher, S. & Guilak, F. Viscoelastic properties of zonal articular chondrocytes measured by atomic force microscopy. *Osteoarthritis Cartilage* **14**, 571-579 (2006).

18. Sokolov, I., Dokukin, M. E. & Guz, N. V. Method for quantitative measurements of the elastic modulus of biological cells in AFM indentation experiments. *Methods* **60**, 202-213 (2013).

19. Efremov, Y. M. *et al.* The effects of confluency on cell mechanical properties. *J. Biomech.* **46**, 1081-1087 (2013).

20. Efremov, Y. M. *et al.* Mechanical properties of fibroblasts depend on level of cancer transformation. *BBA-Mol. Cell Res.* **1843**, 1013-1019 (2014).

21. Zhang, L., Yang, F., Cai, J. Y., Yang, P. H. & Liang, Z. H. In-situ detection of resveratrol inhibition effect on epidermal growth factor receptor of living MCF-7 cells by Atomic Force Microscopy. *Biosens. Bioelectron.* **56**, 271-277 (2014).

22. Celik, E., Abdulreda, M. H., Maiguel, D., Li, J. & Moy, V. T. Rearrangement of microtubule network under biochemical and mechanical stimulations. *Methods* **60**, 195-201 (2013).

23. Hertz, H. On the contact of elastic solids. *J. Reine Angew. Math*. **92**, 110 (1881).

24. Timoshenko, S. P. & Goodier, J. Theory of elasticity. *Int. J. Bulk Solids Storage Silos.* **1** (2014).

25. Harris, A. R. & Charras, G. Experimental validation of atomic force microscopy-based cell elasticity measurements. *Nanotechnology* **22**, 345102 (2011).

26. Nijenhuis, N., Zhao, X., Carisey, A., Ballestrem, C. & Derby, B. Combining AFM and acoustic probes to reveal changes in the elastic stiffness tensor of living cells. *Biophys. J.* **107**, 1502- 1512 (2014).

27. Kirmizis, D. & Logothetidis, S. Atomic force microscopy probing in the measurement of cell mechanics. *Int. J. Nanomed.* **5**, 137-145 (2010).

28. Dimitriadis, E. K., Horkay, F., Maresca, J., Kachar, B. & Chadwick, R. S. Determination of elastic moduli of thin layers of soft material using the atomic force microscope. *Biophys. J.* **82**, 2798-2810 (2002).

29. Puech, P. H. *et al.* Measuring cell adhesion forces of primary gastrulating cells from zebrafish using atomic force microscopy. *J. Cell Sci.* **118**, 4199-4206 (2005).

30. Gao, H. & Yao, H. Shape insensitive optimal adhesion of nanoscale fibrillar structures. *Proc. Natl. Acad. Sci. USA.* **101**, 7851-7856 (2004).
